# Recovery After Nontraumatic Lower Limb Amputation: A Scoping Review of Longitudinal Research Studies

**DOI:** 10.1016/j.arrct.2026.100671

**Published:** 2026-07-02

**Authors:** Szu-Ping Lee, Fateme Eskandarighargheh, Faiza Magsi, Chloe Akiona-Bannan, Jocelyn Contreras, Kayla Cheyenne Francisco, Kentaro Lee, Xan Goodman, Jenny Kent, Sheila Clemens

**Affiliations:** a Interdisciplinary Health Science PhD Program, School of Integrated Health Science, University of Nevada, Las Vegas, NV; b Department of Physical Therapy, University of Nevada, Las Vegas, NV; c Lied Library, University of Nevada, Las Vegas, NV; d Department of Physical Therapy, University of Kentucky, Lexington, KY

**Keywords:** Lower limb amputation, Longitudinal, Outcome measures, Physical, Psychological, Rehabilitation

## Abstract

**Objective:**

Longitudinal research studies are essential for understanding the time course of functional recovery after lower limb amputation (LLA). The purpose of this scoping review was to evaluate the existing evidence base regarding midterm to long-term outcomes following nontraumatic LLA.

**Data Sources:**

A scoping review was conducted per the Joanna Briggs Institute and Open Science Framework guidelines. Based on purpose, 7 databases (PubMed, CINAHL, Embase, Scopus, Web of Science, SportDiscus, and Google Scholar) were searched for studies published between 2000 and 2024, in English, Mandarin Chinese, Tagalog, Persian, or Spanish.

**Study Selection:**

Eligible studies must include ≥50% of participants with nontraumatic major LLA within 3 years after their amputation and report outcomes at ≥2 time points spanning ≥3 months.

**Data Extraction:**

We extracted data including study design, phase of rehabilitation, duration of follow-up, participant characteristics, outcome measures used, and intervention purpose and types.

**Data Synthesis:**

Of 5936 records screened, 85 were included in this review. Most studies were conducted in Europe (42.4%), with much less representation from Asia, Oceania/Australia/New Zealand, Africa, and Latin America. The studies encompass data from a total of 424,422 participants (mean age: 58.3y; 55% men). Specific to time since amputation, 57 studies (67%) started immediately after amputation, and only 21% of studies followed patients beyond 1 year postamputation. The most commonly reported outcome domains are performance-based (19.5%), psychological (18.3%), and other medical outcomes (17.2%). Of the 174 unique outcome measures identified, 55.7% are patient-reported, and 44.2% are performance-based measures.

**Conclusions:**

This review highlights a growing body of evidence regarding longitudinal recovery after nontraumatic LLA. The current evidence base focuses primarily on the perioperative phase. Identified gaps in research include disparity in geographical and population representation, lack of long-term follow-up, and lack of participation outcomes such as social reintegration and returning to work.

## Highlights

• This scoping review synthesized 85 longitudinal studies on recovery after nontraumatic lower limb amputation published between 2000 and 2024.
• Most studies were conducted in Europe (42.4%) and North America (40%), highlighting a lack of representation from Asia, Africa, and Latin America.
• The majority of research focused on early (perioperative) recovery, with only 20% of studies following participants beyond 1 year postamputation.
• Of the 174 distinct outcome measures identified, 55.7% are patient-reported and 44.2% are performance-based, covering a wide range of outcomes.
• Major gaps identified include limited long-term follow-up, underreporting of reintegration outcomes, and insufficient inclusion of socioeconomic and racial/ethnic diversity.

Lower limb amputation (LLA) is a life-altering event that profoundly disrupts patients’ physical function, psychological well-being, and overall quality of life.^1^ Causes of LLA are broadly categorized as traumatic or nontraumatic. Traumatic amputations are typically caused by combat, motor vehicle, or occupational injuries. Nontraumatic amputations are typically medical procedures performed to reduce the spread of infection or tissue necrosis from dysvascular diseases such as diabetes and peripheral arterial disease.^2^ Although the causes and prevalence of LLA differ, in most developed countries, roughly 75% of LLA are nontraumatic due to diabetes and/or peripheral arterial disease in older individuals,^3^, ^4^, ^5^ whereas LLAs in underdeveloped countries are more often due to trauma.^6^, ^7^, ^8^, ^9^ The current increasing prevalence of nontraumatic LLA in the United States is driven by increases in chronic diabetes and other dysvascular diseases in younger age groups (<65y).^5^

Over 1 million people live with major LLA (defined as losing at least the ankle at the talocrural joint, excluding toe amputations), and about 150,000 people undergo an LLA procedure each year in the United States.^6^ These individuals often experience significant disability,^2^^,^^7^ including changes in physical and psychological well-being and functioning, which contribute to disabilities such as pain, increased fall risk, and reduced social participation.^7^ In addition, prosthetic adaptation takes time; issues related to prosthesis use, such as changes in residual limb volume and residual limb pain, are aspects of the natural recovery process after LLA and may impede mobility functions initially.^2^ Although the severity of these symptoms usually improves over time, they still significantly affect the quality of life of individuals with LLA,^8^ particularly during the first months and years after LLA.

As a life-saving procedure, the clinical decision to amputate heavily depends on whether the patient’s quality of life and overall health would improve after surgery. LLA affects quality of life in many ways and may lead to reduced participation, such as difficulty in returning to work and limitations in serving other life roles. For example, the window for regaining ambulatory function is typically within 1-2 years after LLA.^9^ Chronic pain conditions, such as phantom limb pain and residual limb pain,^10^ and depression and anxiety,^11^ are highly prevalent following LLA and may persist to interfere with functional recovery.^12^ Despite the need to understand the time course of physical and psychological rehabilitation in this growing clinical population, a comprehensive review of the current evidence regarding mid to long-term changes of outcomes after nontraumatic LLA has not been conducted.

To address this critical gap, we conducted this scoping review with a specific focus on mid- to long-term recovery (defined as 3mo or longer) after nontraumatic LLA. Specifically, we sought to answer the research question: “How have changes in physical and psychological outcomes after nontraumatic LLA been studied?” with the following 2 subquestions: (1) “How have outcome measures been reported in longitudinal studies for adults with nontraumatic LLAs?” and (2) “What rehabilitation interventions have been investigated to improve outcomes after nontraumatic LLA over time?”

## Methods

This scoping review was conducted in accordance with the guidelines from the Joanna Briggs Institute^13^ and Preferred Reporting Items for Systematic reviews and Meta-Analyses Extension for Scoping Reviews.^14^ Our research team used the PCC framework (population, concept, and context) to define the scope of this review based on our research questions. The review protocol was registered with the Open Science Framework under registration ID: DOI 10.17605/OSF.IO/W5KS6.

### Eligibility criteria

#### Population

The population studied in this scoping review included adults aged ≥18 years of any sex with a major LLA (defined as losing at least the ankle at the talocrural joint) due to nontraumatic causes, regardless of prosthetic use status. We focused on amputations that result from nontraumatic causes, such as diabetes and dysvascular diseases but recognized that most research studies on the LLA population were most likely to include a mixture of individuals with traumatic and nontraumatic amputation. Therefore, we operationally decided to include studies that reported participants’ cause of amputation if ≥50% of them were of the nontraumatic causes. Furthermore, this review was limited to studies focusing on adults, because recovery following congenital limb difference and congenital limb absence is likely different from acquired limb loss later in life.

#### Concept

Outcome measures are any characteristic or quality measured to assess a patient’s physical and psychological functioning, collected consecutively during a longitudinal study (defined as measuring participants’ outcomes at least twice over a period of ≥3mo). Furthermore, we focused on the operationally defined mid- (3-6mo) to long-term (˃6mo) recovery of function starting within 3 years from the amputation surgery. We focused on these first years as it is a critical period to understand patients’ physical and psychological symptoms and adaptations after LLA.

#### Context

The context of this scoping review was broadly defined to allow a maximum exploration of the literature, to include all articles with consecutive reporting of longitudinal outcomes in adults with nontraumatic LLA. Based on the languages researchers of this study spoke, the included articles had to be available in either English, Mandarin Chinese, Tagalog, Persian, or Spanish.

To understand the characteristics of the participants of the research studies, we focused on reported participant characteristics, including etiology of the amputation, time since amputation, sex and/or gender, socioeconomic status, race, culture, ethnicity, participation in familial and professional settings, as well as the geographic location where the study was conducted.

We identified studies with longitudinal designs, time-series, prospective and retrospective cohorts, case-control studies, and other study designs that consecutively report participant outcomes over rehabilitation and life-long timeframes. Studies were categorized into 5 phases of rehabilitation: preoperative, postoperative, preprosthetic, prosthetic, and maintenance/life-long care. The preoperative phase was designated as when any data collection happened before the amputation surgery; if any data collection was conducted after the surgery within the inpatient period, it was considered postoperative. Preprosthetic phase was defined as the period following surgery but before the fitting of a prosthesis, during which patients typically undergo conditioning, residual limb shaping, and preparatory rehabilitation. The prosthetic phase referred to the period in which individuals were being fitted for and learning to use their prosthetic device, including gait training and prosthesis adjustments. The maintenance/long-term care was defined as data collection after the previous phases, with a follow-up of >2 years after LLA. A study may encompass more than one phase of rehabilitation.

In addition to the study design types mentioned earlier, qualitative studies and case reports/series were also considered, given that they may provide consecutively reported qualitative data, including self-reported outcomes and interviews. Review and opinion papers without primary data were excluded. Conference abstracts that do not provide the required information were also excluded.

### Search and screening strategy

Tailored search strategies were developed, along with the help of a research librarian (X.G.), to capture relevant studies in 7 databases: Medline (PubMed), CINAHL, Embase, Google Scholar, Scopus, SPORTDiscus, and Web of Science Core Collection. The search strategies, including all identified keywords (see supplemental appendix S1) and index terms, were adapted for each included database and/or information source. To ensure the validity of our search strategy before conducting the actual search, 10 articles that were previously identified to match our inclusion criteria were used to test the search strings. Each search string was considered acceptable only if it retrieved >80% of those articles that were confirmed to be indexed in a given database. The Google Scholar search was conducted using a set of broad search terms to ensure no relevant studies were missed by the queries of other databases. Furthermore, the reference lists of all included sources of evidence were screened for additional studies. This process was applied to all included papers and was considered complete when no new relevant papers were identified. Studies published after January 2000 up to the search date were included.

The final search was conducted on April 6, 2024. An additional hand search was conducted at the end of the data extraction process to capture articles published by the end of 2024. Following the search, all identified citations were collated and uploaded into EndNote to remove duplicates; remaining records were then exported to Covidence systematic review software^a^ (Veritas Health Innovation).

The following criteria were used during the screening process: (1) the study collected primary data; (2) the studied population contained adults with LLA; (3) cause of amputation in ≥50% of the study participants was nontraumatic; (4) the study reported time after amputation for the participants, and the average time was <3 years at the time of the data collection; (5) the study reported patient outcomes measured at least twice over a period of ≥3 months (from the first to the last assessment); (6) the study was published between 2000 and 2024; and (7) the full text is available in English, Mandarin Chinese, Tagalog, Persian, or Spanish. Detailed screening tool and reasons for exclusion can be found in supplemental appendix S2.

**Fig 1:**
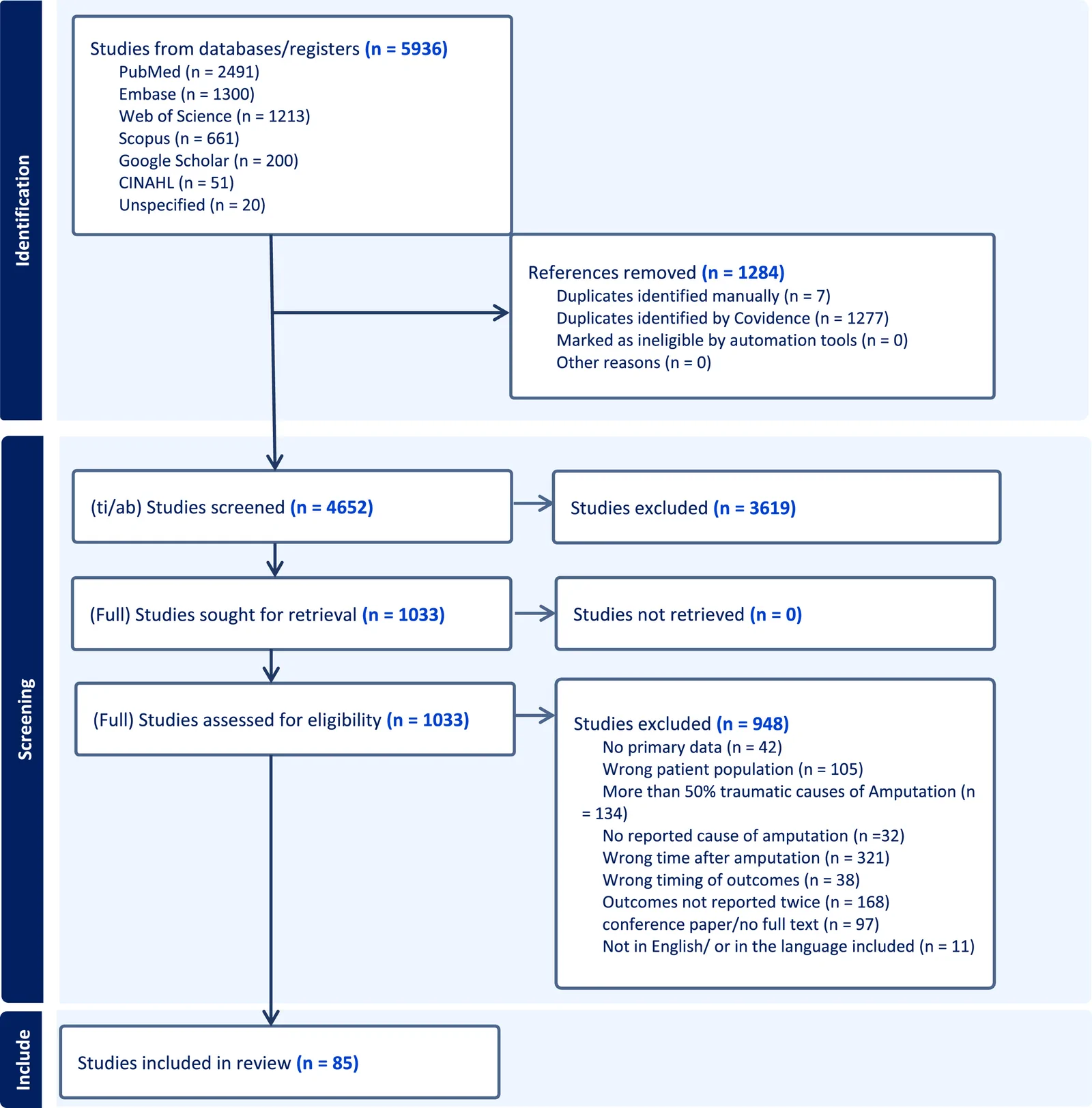
PRISMA-ScR flowchart of the review process. The stepwise process of screening the articles and the potential reasons for their exclusions.

Two independent reviewers assessed the titles and abstracts of the search results for potential eligibility. Studies that met the inclusion criteria were then subjected to a full-text review by 2 reviewers. An additional reviewer resolved any disagreements that arose between the 2 reviewers at each stage of the selection process. The results of the search and the study inclusion process were reported in a Preferred Reporting Items for Systematic Reviews and Meta-analyses extension for scoping review flow diagram (fig 1).^14^

### Data extraction

Two independent reviewers extracted data from papers included in the scoping review using a data extraction tool developed by the reviewers (supplemental appendix S2). Data on participant characteristics, study designs and settings, methods, follow-up time points, intervention, outcome measures, and key findings were extracted. A third reviewer provided the consensus on any differences between the 2 data extraction sheets. Any disagreements that occurred between reviewers were resolved through discussion. Researchers contacted authors of papers to request missing full texts when necessary.

## Results

### Study selection

A total of 5936 records were identified through database searches. After removing 1284 duplicates, 4652 records remained for title and abstract screening. Of these, 1033 full-text articles were assessed for eligibility. Ultimately, 85 studies met the inclusion criteria and were included in the data extraction (fig 1).

### Geographic distribution

All included studies were published in English. The majority were conducted in Europe (42.4%) and North America (40%), with smaller contributions from Asia, Oceania, Africa, and Central/South America (table 1).

**Table 1:** Geographic location of the included studies by continent

Continent	Count (%)
Europe	36 (42.4)
North America	34 (40)
Asia	5 (5.9)
Oceania	4 (4.7)
Africa	3 (3.5)
Central/South America	2 (2.3)
Multicontinental	1 (1.2)

### Demographics

Across the 85 included studies, there were a total of 424,422 participants. Of these, 233,289 (54.96%) were men, and 191,133 (45.09%) were women, with a mean age of 58.3 years. Most amputations were due to nontraumatic causes (98.6%), with diabetes (44.3%) and peripheral vascular disease (34.46%) being the most commonly reported etiologies (table 2).

**Table 2:** Characteristics of participants in the included studies

Category	Count (%)
Total participants	424,422
Men	233,289 (54.96%)
Women	191,133 (45.09%)
Mean age (y)	58.3
Nontraumatic cause	98.6%
Diabetes	11,484 (44.3%)
Peripheral vascular disease	8,927 (34.46%)
Cancer	173 (0.67%)
Transfemoral	7305 (44.90%)
Transtibial	6951 (42.79%)
Ankle disarticulation	1476 (9.09%)
Bilateral	326 (2.00%)
Knee disarticulation	114 (0.70%)
Hip disarticulation	69 (0.40%)

### Level of amputation

Among the study participants, the most frequently reported amputation levels were unilateral transfemoral (44.90%) and unilateral transtibial (42.79%), followed by less common levels such as ankle disarticulation (9.09%) and bilateral amputation (2%; table 2).

### Analysis of study exclusion criteria

The most reported exclusion criterion was the presence of cognitive or communication impairments (41.2%), including inability to provide informed consent, language barriers, and cognitive deficits. Other frequently reported exclusions included comorbid diseases (16.4%), traumatic amputations (10.5%), and partial foot/toe amputations (4.7%).

### Reporting of participant characteristics

**Fig 2:**
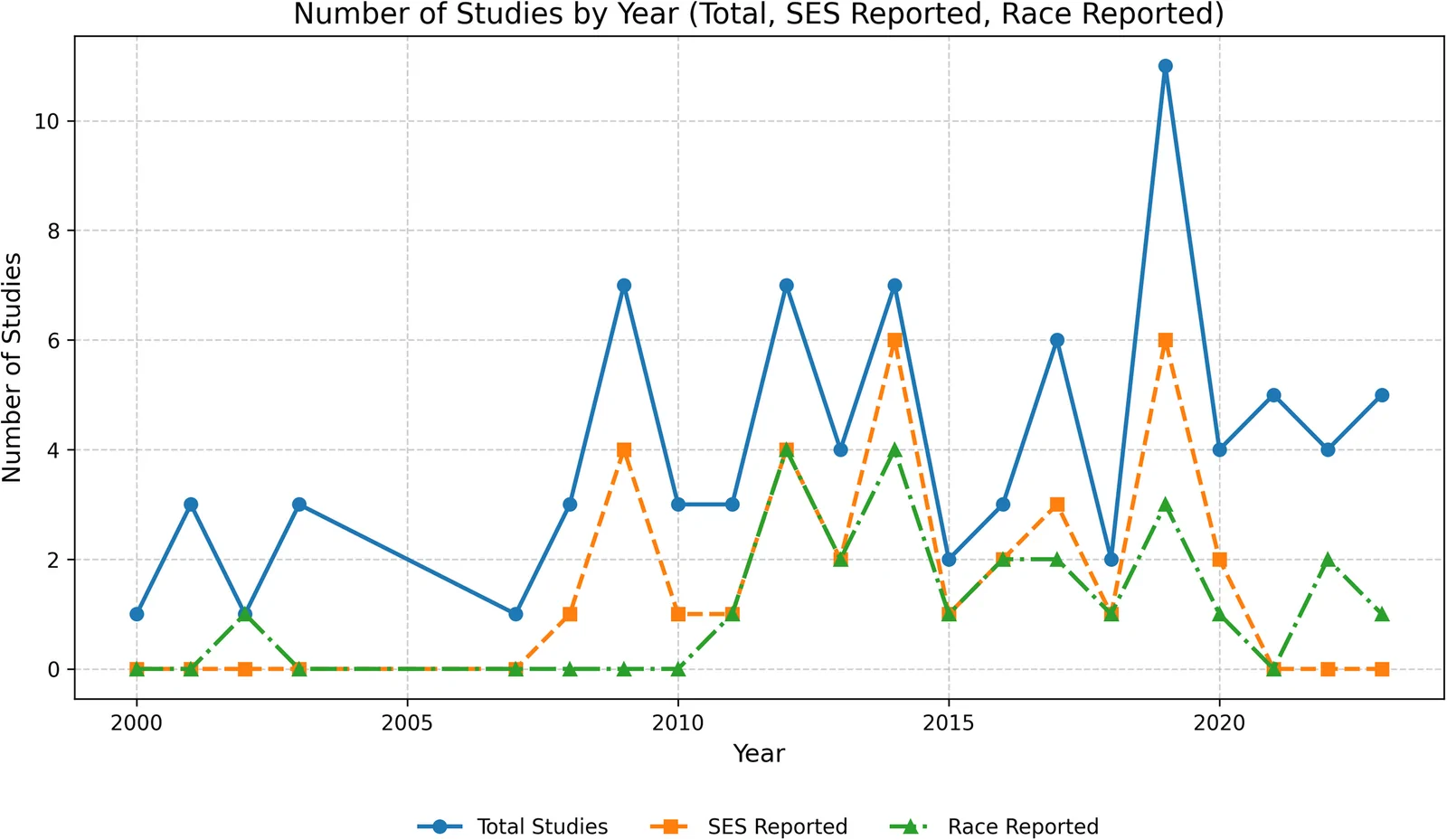
Time trend of studies reporting participant socioeconomic/race information. The visual representation of reporting socioeconomic and race information over the years among the included articles. SES,

Of the studies included, 25 (29.41%) reported the race and/or ethnicity of their study participants. The majority of them (22 out of 25) were conducted in North America. The remaining 3 studies were distributed across Africa (1), Europe (1), and Oceania (1). No studies conducted in Asia or Central and South America reported race or ethnicity. A range of socioeconomic characteristics has been reported with variable frequency (table 3). Further analysis showed that among the 52 studies that were published within the last 10 years (2013-2023; fig 2), 23 (44.23%) reported socioeconomic status and 19 (36.54%) reported race and/or ethnicity. In contrast, of the 33 studies published earlier between 2000 and 2012, only 10 studies (30.30%) reported socioeconomic status and 6 studies (18.18%) reported race and/or ethnicity (fig 2).

**Table 3:** Socioeconomic characteristics reported

Variable	Count (%)
Education	20 (60.6)
Marital status	17 (51.5)
Living situation/residence	15 (45.5)
Employment/retirement status	11 (33.3)
Income	10 (30.3)
Social/family support	4 (12.1)
Insurance coverage	3 (9.1)
Smoking history	1 (3.0)
Mode of transportation	1 (3.0)

### Study design and geographical location

The most common experimental design of the included studies is the cohort study design (68.2%), with a smaller proportion of randomized controlled trials (14.1%), case reports (10.6%), nonrandomized experimental trials (3.5%), qualitative studies (2.3%), and cross-sectional studies (1.2%). The majority of the cohort studies and randomized controlled trials were prospective in nature and from North America (30, 35.3%) and Europe (30, 35.3%), together accounting for more than two-thirds of the included articles. North American studies were primarily from the United States (25) and Canada (5), whereas European contributions included the Netherlands (8), the United Kingdom (5), Scotland (4), and Italy (3). Representation from Asia (3, 3.5%), Africa (3, 3.5%), and Oceania (3, 3.5%) was limited. One study did not specify the country of origin.

### Phase of rehabilitation

Most studies started data collection in the preoperative and postoperative phases of rehabilitation (45.8 %). Only 7 studies covered the prosthetic phase, and only 1 study focused on the maintenance/life-long care phase. No study started data collection during the preprosthetic phase of rehabilitation. Specific to time since amputation, 57 studies (67.0%) started immediately after amputation (ie, typically in an inpatient setting), and a further 17 studies (20%) reported data collection beginning within a time frame of 1 week to 6 months postamputation. The remaining 11 studies (13.7%) included participants with a time since amputation greater than 6 months.

### Study settings and follow-up duration

**Fig 3:**
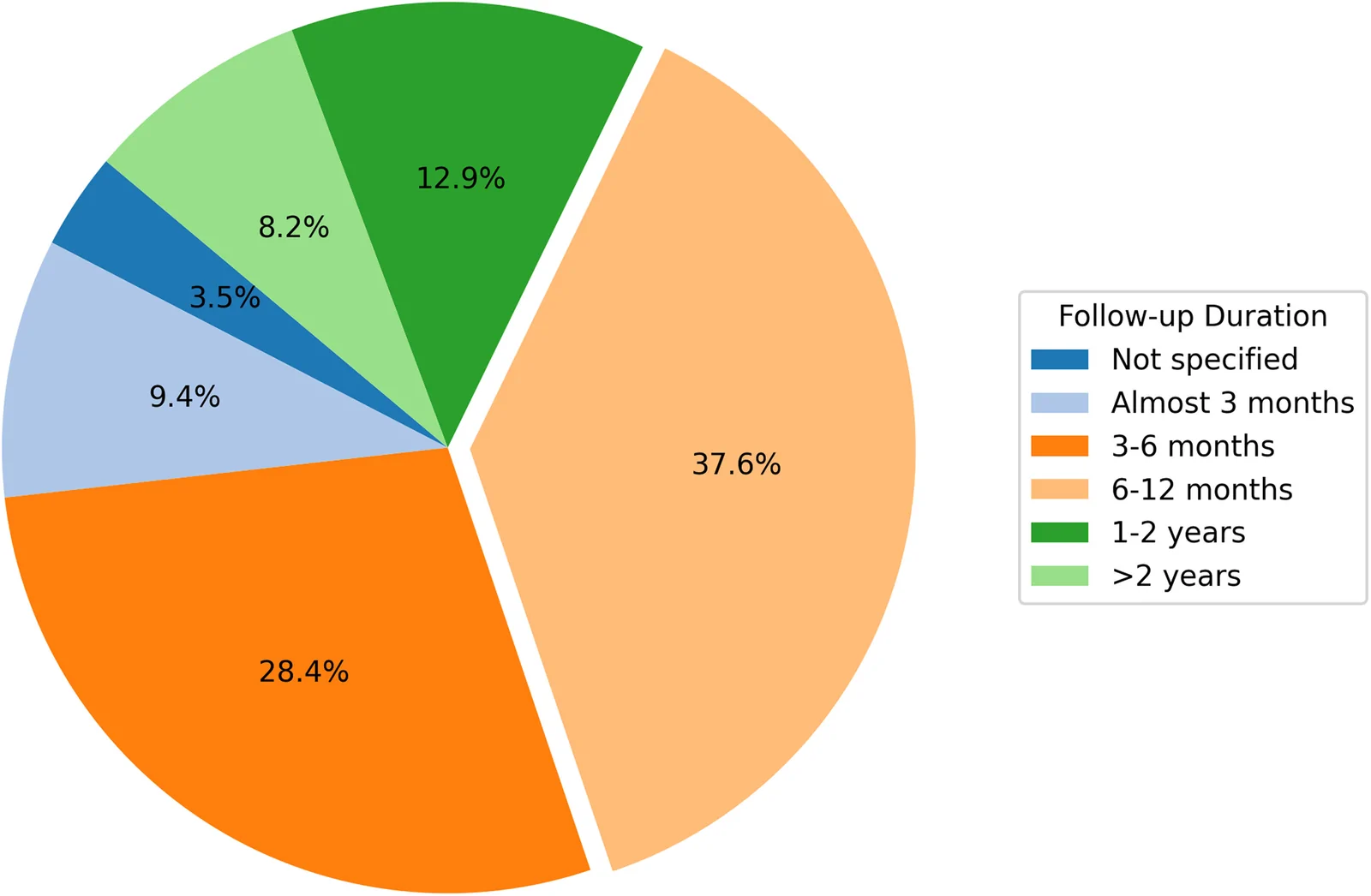
Follow-up durations. Pie chart showing the proportion of participants by follow-up duration. The largest slice represents participants followed for 6-12 months. Percentages are indicated on the slices.

The included studies were conducted in a range of rehabilitation environments. The most common setting was inpatient (91.8%), followed by outpatient (70.6%), home/other living facilities (42.4%), and lab-based settings (3.5%). The follow-up durations varied greatly. The most common follow-up duration was 6-12 months (37.6%; fig 3). Only 8.2 % of the studies followed the participants beyond 2 years (fig 3). Follow-up frequency and duration for each study were summarized in supplemental appendix S3.

### Intervention types

Seventeen (20%) of the articles were observational without any interventions as part of the study. Thirty-three studies (48.5%) implemented standard care, including rehabilitation, which typically involved multidisciplinary care such as physical therapy and occupational therapy. Out of the 35 studies with specific interventions, surgical and medical (65.7%), prosthetic fitting and training (17.1%), and home-based programs (17.1%) were the emergent types of intervention investigated (table 4).

**Table 4:** Types of interventions

Intervention Type	Count (%)
Surgical/medical (eg, TMR, flap, PNC)	23 (65.7)
Prosthetic fitting and training	6 (17.1)
Home-based programs (eg, acupuncture, physical therapy, and so forth)	6 (17.1)

*Abbreviations:* PNC, peripheral nerve catheter; TMR, targeted muscle reinnervation.

### Outcome measures

**Fig 4:**
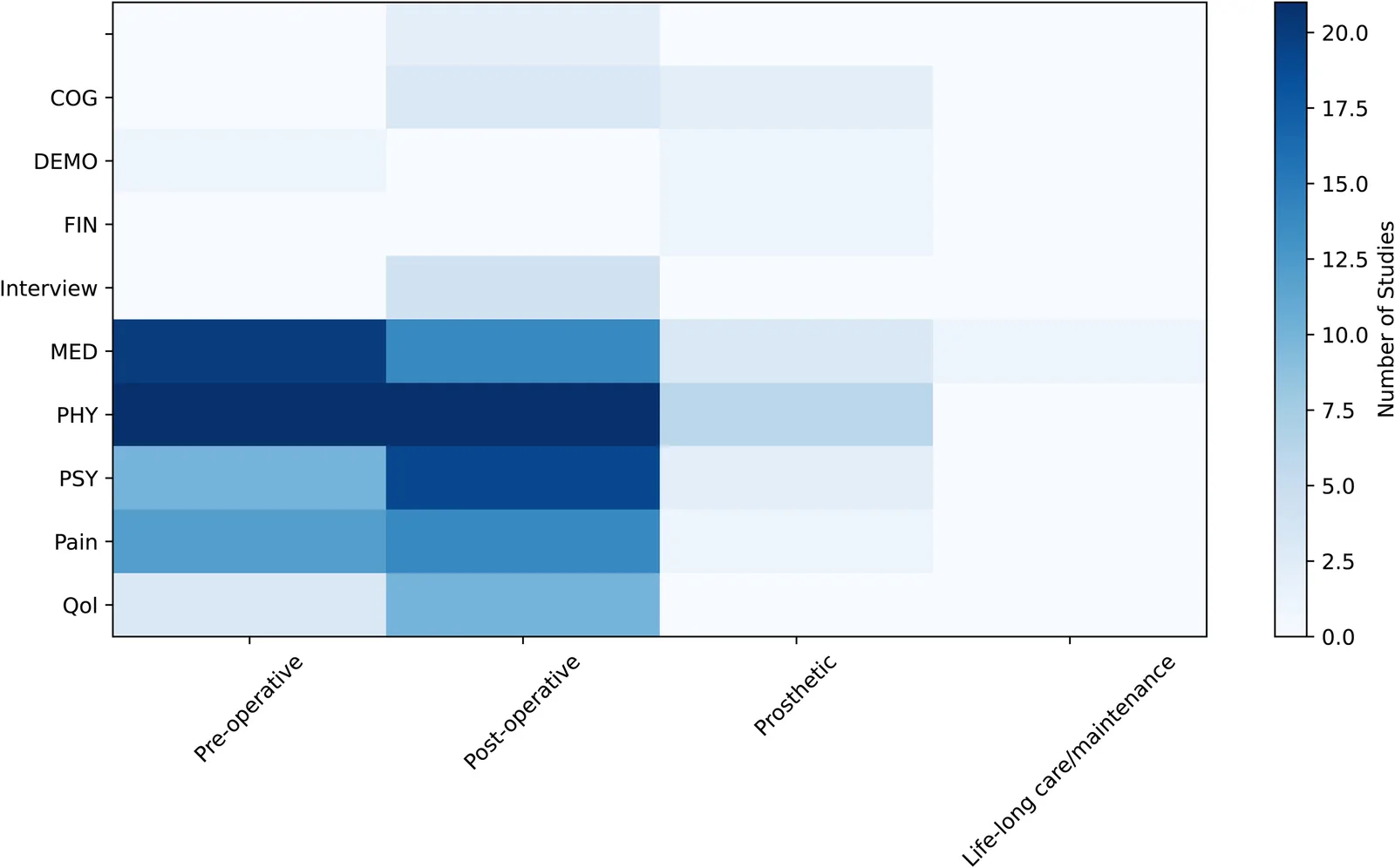
Heatmap of study numbers versus types of outcome measures and phase of rehabilitation at the start of data collection. Usage of different kinds of outcome measures across the phases of rehabilitation, and the number of studies among the included articles. Abbreviations: COG, cognitive outcome measures; DEMO, demographic outcome measures; FIN, financial outcome measures; MED, medical outcome measures; PHY, physical outcome measures; PSY, psychological outcome measures; QoL, quality of life outcome measures.

Across the 85 included studies, we identified 174 distinct outcome measures, comprising 97 Patient-Reported Outcome Measures and 77 Performance-Based Outcome Measures. A comprehensive list of these measures is provided in supplemental appendix S4. Performance-based physical outcome type had the largest number of measures, followed by psychological and medical outcome types (table 5). Only 2 studies contained a qualitative assessment of patient perception.^15^^,^^16^ The qualitative findings were typically not used to infer quantitative outcomes. Pain and its related properties, such as intensity, expectation, and satisfaction, were also commonly assessed using various pain measures, resulting in a total of 25 outcome measures. Cognitive function, financial cost, and participation are some of the less-reported outcome domains. We further analyzed the outcome measures based on the studied phase of rehabilitation. A heatmap was generated to provide a visualization of the outcome measures used in different phases of rehabilitation (fig 4). Performance-Based Outcome Measures for physical performance were the most commonly used outcome measures during the preoperative and postoperative phases of rehabilitation. A wide range of medical outcome measures are commonly utilized in studies that initiate data collection during the preoperative phase (these studies often extend into the postoperative phase). Selection of outcome measures in this phase largely depends on the research questions; measures of wound healing, comorbidities (eg, Charlson Comorbidity Index), and prescription drug use are the most ubiquitous in this type of study.

**Table 5:** Outcome domains and measures with the examples from included studies

Measure Categories	Examples	Number of Outcome Measures	Study Example
Cognitive	Validity of Cognition, Trail Making Test	8	Rostaminejad et al^17^
Demographics	Type of Residence Before Amputation, Office of Population Censuses and Surveys Scale	2	Panesar et al^18^
Financial	Average Costs of Prosthetics	1	Rommers et al^19^
Medical	Veterans Administration Large Health Survey, Time of First Dose of Analgesia After Surgery	30	Czerniecki et al^20^
Pain	Visual Analog Scale, Self-Reported Leeds Assessment of Neuropathic Symptoms and Signs Tool	25	Bakeer et al^21^
PBOM-Physical	Timed Up and Go, Walking Speed	34	Christiansen et al^22^
PROM-Physical	World Health Organization Disability Assessment Schedule 2.0, Walking Ability Index	29	Coffey et al^23^
Psychological	Trinity Amputation and Prosthesis Experience Scales TAPES (complete or subscale), The Groningen Questionnaire: Problems After Arm Amputation	32	Norvell et al^24^
Quality of life	World Health Organization Quality of Life Scale Brief Version, The Euroqol-5D	13	Godlwana et al^25^
Participation	Trinity Amputation and Prosthesis Experience Scales, the Questionnaire for Persons with a Transfemoral Amputation, the Late Life Disability Instrument	11	Akhtar et al^26^

Abbreviations: PBOM, Performance-Based Outcome Measure; PROM, Patient-Reported Outcome Measures.

## Discussion

This scoping review comprehensively examined studies that investigated mid- to long-term rehabilitation outcomes in individuals after nontraumatic LLA. Of the 85 studies included, we found that both sexes are represented (55% men, 45% women). However, there exists an inequity in the geographical representation of research. Europe, representing just 9% of the global population,^27^ contributed the largest proportion of studies. Notably, Scotland and the Netherlands demonstrated high research density relative to their population sizes. North America, particularly the United States, accounted for 40% of the studies, despite comprising only 7.5% of the world population. In stark contrast, Asia and Africa, home to over 60% and 18.7% of the global population, were severely underrepresented, contributing fewer than 6% and 3.5% of studies. Even populous countries like China and India had minimal representation. These disparities likely reflect limited research resources, different research priorities, stigma to amputation, language barriers in publication, and other systemic reasons of exclusion from global research networks. Such inequities limit the generalizability of findings and highlight the urgent need to support research efforts in underrepresented regions.

Examining the reported participant characteristics, we found that socioeconomic characteristics (eg, income, education level, insurance status, and so forth) of participants were reported in fewer than 40% of studies, most commonly in North American and European studies, whereas studies from Asia, Africa, and Latin America rarely report them. Furthermore, race and ethnicity data were reported infrequently (in only 29% of the included studies). Among studies that reported race/ethnicity, almost all were from North America (88%), very few from Europe (4%), and virtually none from other global regions, despite the fact that the vast majority of the world’s racial and ethnic diversity exists outside of North America.^28^ This is a potential issue as race/ethnicity and related disparities have been shown to be important predictors for both the risk and outcomes of amputation.^29^ The lack of reporting key socioeconomic variables indicates a knowledge gap in understanding how health disparities and social determinants of health affect functional recovery after LLA.^14^, ^30^ However, we observed signs of improvement; between 2000-2012 and 2013-2023, the proportion of studies reporting race/ethnicity rose from 18.2% to 36.5%, and studies reporting socioeconomic status increased from 30.3% to 44.2%, respectively. This promising trend indicates a growing recognition of the complex environmental and psychosocial contributions to disability and health, though continued progress is needed.

Although it is widely recognized that recovery after LLA takes time, the follow-up durations of the included studies were generally limited, with the majority of studies focusing on outcomes within only the first-year postamputation, primarily between 3-6 months (28.4%) and 6-12 months (37.6%). Use of a prosthetic leg for restoration of ambulation is often a primary goal of patients after LLA, and prosthetic training typically does not begin sooner than 3 months postamputation. However, complications due to comorbidities, surgical healing rates, or financial difficulties in prosthetic provision can often significantly delay prosthetic training in individuals with nontraumatic LLA, increasing potential physical and psychological impacts on functional recovery and quality of life.^31^ Yet, the majority of the studies in this review occurred during the perioperative phases of care (preoperative and immediately postoperative), with long-term follow-up beyond 2 years being rare. This finding is profound because many higher-level functional outcomes, such as social reintegration and participation, typically take longer to accomplish and can have a lasting impact on patients’ quality of life after LLA.^32^ Prospective longitudinal research to track changes in long-term outcomes, and to examine which biopsychosocial factors are associated with better outcomes, is lacking in amputation research. Specifically, there is a dearth of prospective research on the lived experiences of people with LLA and how perceptions of environment, culture, and society may shape recovery over time. This results in many unknowns, making it difficult to develop effective, comprehensive, patient-centric interventions to optimally address the full range of recovery after LLA.

The majority of included studies were prospective in design, including both longitudinal cohort studies with follow-ups and randomized controlled trials (70/85, 82.3%). The high percentage of these 2 designs indicates a relatively high-quality level of the available evidence in this area of research. The focus on prospective methodologies allows for clearer temporal relationships and reduces certain biases compared with retrospective designs. Notably, these prospective studies were predominantly conducted in North America and Europe, suggesting that high-quality, forward-looking research in this field is concentrated in these regions. The limited representation from Asia, Africa, and Oceania highlights a potential gap in prospective evidence globally and underscores the need for more regionally diverse investigation to address recovery after LLA in different contexts.

Studies examining specific interventions accounted for 41% of the included studies. Standard medical and surgical care accounted for the majority of these interventions (48.5%). Other rehabilitation approaches, such as prosthetic training and home-based programs, accounted for 34.2% of the interventional studies. Our findings highlighted a notable absence of studies focusing on psychosocial interventions, such as the effects of peer support and vocational rehabilitation in this population, despite clear indications for this need after LLA.^33^ This is likely because these interventions have been primarily studied cross-sectionally and/or have not been examined soon after amputation (ie, within 3y). These understudied psychosocial constructs, such as self-efficacy, income, and social support, are strongly associated with functional mobility in the LLA population^14^ and offer potential avenues for interventions to improve life after amputation. Future research should focus on how psychosocial interventions benefit long-term physical and psychological recovery, as well as higher levels of functional outcomes such as returning to work. This may be particularly important in the coming years due to the increasing number of working-age individuals with diabetes-related LLA in countries such as the United States.^34^

The identified measures spanned a broad range of outcome domains, including physical mobility, pain, psychological, quality of life, and cognitive function. Physical mobility outcomes were commonly evaluated (63 measures), utilizing both performance-based measures (eg, gait velocity, 6-minute walk test, L-test) and patient-reported measures (eg, Locomotor Capabilities Index, Rivermead Mobility Index, and Prosthetic Limb Users Survey of Mobility). Psychological and psychosocial outcomes were addressed using 32 distinct outcome measures, making this one of the more comprehensively assessed domains. Measures included the Patient Health Questionnaire-9, Geriatric Depression Scale, Hospital Anxiety and Depression Scale, and Satisfaction with Life Scale, among others. These instruments, although not specifically designed to measure psychological adjustments after LLA, examined constructs such as depression, anxiety, and self-efficacy commonly related to LLA. Incorporating psychological and quality of life outcome measures such as the 36-Item Short Form Health Survey, the 12-Item Short Form Health Survey, the EuroQol 5-Dimension Questionnaire, and the World Health Organization Quality of Life–BREF (WHOQOL-BREF) may capture broader impacts of amputation beyond physical recovery.

Although we recognize that the selection of outcome measures in a study is often based on the research questions, the large variance in outcome measures observed in this study may impair the translation and application of research findings. To improve the quality and consistency of outcome measurement during amputation care, several attempts have been made in recent years. In 2021, the International Society for Prosthetics and Orthotics conducted a series of projects to develop a core dataset (Lower Extremity Amputation Dataset) and a recommended set of outcome measures (Consensus Outcome Measures for Prosthetic and Amputation Services) intended to be used for assessing outcomes in people with lower limb loss before and after rehabilitation care.^35^^,^^36^ The resulting set of core outcome measures is made up of 3 physical performance-based measures (ie, Amputee Mobility Predictor, timed Up and Go test, 2-minute walk test) and 3 patient-reported outcome measures (ie, Prosthesis Evaluation Questionnaire—Utility and Residual Limb Health Subscales, and Trinity Amputation and Prosthesis Experience Scales—Revised). Additional resources, including the US Department of Defense’s Outcome Measures Guidebook for Amputation Care^37^ and the National Institute of Health’s Draft of Common Data Elements for Lower Limb Loss Research^38^ should be consulted when selecting outcome measures in future research.

### Study limitations

Although this scope review was based on a thorough literature search, screening, and rigorous data extraction, this review format has a few limitations. First, no critical appraisal of study quality was conducted due to the highly heterogeneous study designs included. This scoping review prioritized mapping of the current literature to identify critical gaps in the evidence,^14^ which hopefully will lead to future systematic reviews to critically appraise qualities of select types of studies (ie, randomized controlled trials). Second, the mid- to long-term timeframes were operationally defined as 3-6 months and beyond 6 months after amputation, respectively. Although these duration-based timeframes streamlined the process of study screening in this review, we recognize they likely do not apply to all patients with LLA. Third, the synthesis of study findings was limited due to the high degrees of heterogeneity in study design, studied population, and outcome measures reported.

## Conclusion

This scoping review provides a comprehensive examination of the current state of research regarding the mid- to long-term recovery of function after LLA. Critical knowledge gaps include a lack of global population representation, relatively short follow-up duration, and a lack of long-term intervention and outcomes. Studies addressing psychophysical well-being, quality of life, and social determinants of health were identified as potential areas of future research. This study highlights the need for more geographically diverse and multidimensional research to guide holistic, patient-centered rehabilitation for individuals after nontraumatic LLA.

## Supplier

a. Covidence systematic review software; Veritas Health Innovation.

## Disclosure

The authors declare no conflicts of interest related to this study. The research in this work was supported by the Student Opportunity Research Grant from the Department of Physical Therapy, University of Nevada, Las Vegas (UNLV). The research was conducted independently, and no external funding or commercial relationships influenced the design, data collection, analysis, or interpretation of the results. Szu-Ping Lee disclosed a financial relationship with the Encompass Health Corporation and a patent (US patent no. 10335058) that is unrelated to the current work. Xan Goodman disclosed a financial relationship with the National Network of Libraries of Medicine and the Medical Library Association that is unrelated to the current work. Jenny Kent disclosed grants from the Department of Defense and the Nevada IDeA Network of Biomedical Research Excellence that are unrelated to the current work. Sheila Clemens disclosed a grant from the Department of Veterans Affairs, a financial relationship with Bandagist Jan Nielsen in Denmark, travel support from the University of Kentucky, and an unpaid position in the Academy of Federal Physical Therapy that are unrelated to the current work.

## Appendix Supplementary materials

Image, application 1

## Footnotes

Supplementary material associated with this article can be found, in the online version, at doi:10.1016/j.arrct.2026.100671.
