# Supplementary material for: Recovery After Nontraumatic Lower Limb Amputation: A Scoping Review of Longitudinal Research Studies
Source: Arch Rehabil Res Clin Transl. 2026 Jul 2;8(3):100671. doi: 10.1016/j.arrct.2026.100671 (PMC13591168; doi:10.1016/j.arrct.2026.100671)
Supplement: Supplementary file 1 [file mmc1.docx]

**APPENDICES**

### Appendix I: Search strategy from different databases

The search strings are written in order of amputation terms, study design terms, year and language terms, according to every database’s search guide. Please see below for details:

| **1** | **PubMed** | ("Amputees"[MeSH Terms] OR "Artificial Limbs"[MeSH Terms] OR "amputation, surgical"[MeSH Terms] OR "non-traumatic amputation"[Text Word] OR "Amputation Stumps"[MeSH Terms] OR "Bone-Anchored Prosthesis"[MeSH Terms] OR ("below knee amput*"[Title/Abstract] OR "trans tibial amput*"[Title/Abstract] OR "transtibial amput*"[Title/Abstract] OR "through knee amput*"[Title/Abstract] OR "knee disarticulation"[Title/Abstract] OR "trans femoral amput*"[Title/Abstract] OR "transfemoral amput*"[Title/Abstract] OR "above knee amput*"[Title/Abstract] OR "hindquarter amput*"[Title/Abstract] OR "through hip amput*"[Title/Abstract] OR "hip disarticulation"[Title/Abstract] OR "lower limb loss"[Title/Abstract] OR "lower limb prosthe*"[Title/Abstract] OR ("ankle disarticulation"[Title/Abstract] OR "lower limb amput*"[Title/Abstract] OR "ankle disarticulation"[Title/Abstract] OR "lower extremity amput*"[Title/Abstract] OR "transtibial amput*"[Title/Abstract])) OR ("Amputees"[Title/Abstract] OR "Artificial Limbs"[Title/Abstract] OR "amputation surgical"[Title/Abstract] OR "non-traumatic amputation"[Title/Abstract] OR "Amputation Stumps"[Title/Abstract] OR "Bone-Anchored Prosthesis"[Title/Abstract])) AND ("longitudinal studies"[MeSH Terms] OR "longitudinal study"[Title/Abstract] OR "prospective studies"[MeSH Terms] OR "prospective studies"[Title/Abstract] OR "Follow-Up Studies"[MeSH Terms] OR "long-term study"[Title/Abstract] OR "change over time"[Title/Abstract] OR "time series"[Title/Abstract]) AND 2000/01/01:2024/12/31[Date - Publication] |
| --- | --- | --- |
| **2** | **CINAHL** | Box1 TX in all Tex  (((TI "below knee amput*" OR AB "below knee amput*") OR (TI "trans tibial amput*" OR (TI "transtibial amput*" OR AB "transtibial amput*") OR (TI "knee disarticulation" OR AB "knee disarticulation") OR (TI "transfemoral amput*" OR AB "transfemoral amput*") OR (TI "above knee amput*" OR AB "above knee amput*") OR (TI "hindquarter amput*" OR AB "hindquarter amput*") OR (TI "hip disarticulation" OR AB "hip disarticulation") OR (TI "lower limb loss" OR AB "lower limb loss") OR (TI "lower limb prosthe*" OR AB "lower limb prosthe*")) OR ((TI "ankle disarticulation" OR AB "ankle disarticulation") OR (TI "lower limb amput*" OR AB "lower limb amput*") OR (TI "ankle disarticulation" OR AB "ankle disarticulation") OR (TI "lower extremity amput*" OR AB "lower extremity amput*") OR (TI "transtibial amput*" OR AB "transtibial amput*")))  AND  Box 2 TX in all Tex  (MH “longitudinal sturdy”) OR (IT “longitudinal sturdy” OR AB “longitudinal sturdy”) (MH “prospective study”) OR (IT “prospective study” OR AB “prospective study”) OR (MH “follow-up study”) OR (TI “follow-up study” OR AB “follow-up study”) OR (MH “long-term study”) OR (TI “long-term study” OR AB “long-term study”) OR (MH “change over time”) OR (TI “change over time” OR AB “change over time”) OR (MH “time series”) OR (TI “time series” OR AB “time series”) |
| **3** | **Embase** | ('below knee amputation':ti,ab OR 'knee disarticulation':ti,ab OR 'transfemoral amputation':ti,ab OR 'above knee amputation*':ti,ab OR 'hindquarter amputation*':ti,ab OR 'hip disarticulation':ti,ab OR 'lower limb loss':ti,ab OR 'lower limb prosthe*':ti,ab OR 'lower limb amputee':ti,ab OR 'ankle disarticulation':ti,ab OR 'lower extremity amput*':ti,ab OR 'transtibial amputation':ti,ab OR 'bone-anchored prosthesis':ti,ab) AND ('longitudinal study':ti,ab OR 'prospective study':ti,ab OR 'follow-up studies' OR 'long-term study':ti,ab OR 'change over time':ti,ab OR 'time series':ti,ab) AND ([chinese]/lim OR [english]/lim OR [persian]/lim OR [spanish]/lim) AND [2000-2024]/py |
| **4** | **Google Scholar** | "lower limb" AND amputation AND Longitudinal AND “follow up” |
| **5** | **Scopus** | TITLE-ABS ("below knee amput*" OR "transtibial amput*" OR {knee disarticulation} OR "transfemoral amput*" OR "above knee amput*" OR "hindquarter amput*" OR {hip disarticulation} OR {lower limb loss} OR "lower limb prosthe*" ) OR ( "ankle disarticulation" OR "lower limb amput*" OR "ankle disarticulation" OR "lower extremity amput*" OR "transtibial amput*" ) AND TITLE-ABS ( {longitudinal study} OR {follow up} OR {prospective study} OR {time series} OR {long term study} OR {change over time} ) AND PUBYEAR > 2000 AND PUBYEAR < 2024 AND ( LIMIT-TO ( LANGUAGE , "English" ) OR LIMIT-TO ( LANGUAGE , "Chinese" ) OR LIMIT-TO ( LANGUAGE , "Persian" ) OR LIMIT-TO ( LANGUAGE , "Spanish" ) |
| **6** | **SportDiscus** | Box 1 TX All Tex  (((TI "below knee amput*" OR AB "below knee amput*") OR (TI "transtibial amput*" OR AB "transtibial amput*") OR (TI "knee disarticulation" OR AB "knee disarticulation") OR (TI "transfemoral amput*" OR AB "transfemoral amput*") OR (TI "above knee amput*" OR AB "above knee amput*") OR (TI "hindquarter amput*" OR AB "hindquarter amput*") OR (TI "hip disarticulation" OR AB "hip disarticulation") OR (TI "lower limb loss" OR AB "lower limb loss") OR (TI "lower limb prosthe*" OR AB "lower limb prosthe*")) OR ((TI "ankle disarticulation" OR AB "ankle disarticulation") OR (TI "lower limb amput*" OR AB "lower limb amput*") OR (TI "ankle disarticulation" OR AB "ankle disarticulation") OR (TI "lower extremity amput*" OR AB "lower extremity amput*")  AND  Box 2 TX All Tex  (DE “longitudinal sturdy”) OR (IT “longitudinal sturdy” OR AB “longitudinal sturdy”) (DE “prospective study”) OR (IT “prospective study” OR AB “prospective study”) OR (DE “follow-up study”) OR (TI “follow-up study” OR AB “follow-up study”) OR (DE “long-term study”) OR (TI “long-term study” OR AB “long-term study”) OR (DE “change over time”) OR (TI “change over time” OR AB “change over time”) OR (DE “time series”) OR (TI “time series” OR AB “time series”) |
| **7** | **Web of Science** | web of science core collection  TS=(("Bone-Anchored Prosthesis" OR "below knee amputee" OR "below knee amputation" OR "transtibial amputee" OR "transtibial amputation" OR "knee disarticulation" OR "transfemoral amputee" OR "transfemoral amputation" OR "above knee amputee" OR "above knee amputation" OR "hindquarter amputee" OR "hindquarter amputation" OR "through hip amputee" OR "through hip amputation" OR "hip disarticulation" OR "lower limb loss" OR "lower limb prosthetic" OR "ankle disarticulation" OR "lower limb amputee" OR "lower extremity amputation")) AND TS=('longitudinal study' OR 'prospective study' OR 'follow-up studies' OR 'long-term study' OR 'change over time' OR 'time series') AND PY=(2000-2024) AND LA=(English OR Spanish OR Chinese OR Persian) |

### Appendix II: Data Extraction Protocol

1. Review objective: Identify the pattern of mid- to long-term changes in physical and psychological outcomes after amputation surgery

2. Review questions:

How have changes in physical and psychological outcomes after non-traumatic lower limb amputation been studied?

Sub-questions:

a. How have outcome measures been reported in longitudinal studies for adults with non-traumatic lower limb amputations?
b. What interventions have been investigated to improve outcomes after non-traumatic lower limb amputation over time?

3. Method

The methodology of the search and the study inclusion process follow the recommended format of Preferred Reporting Items for Systematic Reviews and Meta-analyses (PRISMA)^18^.

3.1 Participant Inclusion Criteria

**Population**

Type of population (i.e. Veterans Affairs patients, Community Dwelling Adults)

Sample Size

Age

Race and ethnicity composition of the sample

Gender and/or Sex composition of the sample

Socioeconomic status

**Amputation-related information**

Level (hip disarticulation, transfemoral, knee disarticulation, transtibial, ankle disarticulation)

Etiology (% of traumatic vs. non-traumatic, % of dysvascular, infection, cancer, congenital)

Time Since Amputation (average and range)

Duration of follow-up (average and range)

**Concept**

Study design (case study/series, observational [medical chart review], case-control, longitudinal, intervention trial…etc.)

Study setting (inpatient, outpatient, research lab-based, community-based…etc.)

List of Outcome Measures Used

Studies that fulfill all of the following criteria are to be included:

1. The study collected primary data (review or re-analysis such as systematic review, scoping review, literature review, or narrative and comments are excluded).
2. The studied population contained adults with lower limb amputation.
3. Cause of amputation in at least 50% of the study participants was non-traumatic.
4. The study reported time after amputation for the participants, and the average time is less than 3 years.
5. The study reported some forms of patient outcomes measured at least twice over a period of at least 3 months (from the first to the last assessment in the study).
6. The study was published between 2000 and 2024, and the full-text is available in English, Mandarin Chinese, Tagalog, Persian, or Spanish.

Studies that meet one or more of the following are excluded:

1. Published in languages other than English, Mandarin Chinese, Tagalog, Persian, or Spanish
2. Non-primary research
3. Included only children or more than 50% children
4. No reported cause of amputation
5. No reported duration after amputation
6. No measurement of physical or psychological outcomes
7. No consecutive measurement of outcomes that span at least a 3-month period

Appendix III: Complete included studies with follow-up table

| **Study ID** | **Title** | **Type of Study** | **Phase(s) of Rehabilitation** | **Follow-up Frequencies** | **Follow-up Duration** |
| --- | --- | --- | --- | --- | --- |
| **Panesar 2001** | A comparison of three measures of progress in early lower limb amputee rehabilitation. | Cohort | Pre-operative | 3 time points | 3 mo |
| **Bakeer 2023** | The incidence of post amputation syndrome after pericapsular nerve group block versus sciatico-femoral nerve block in patients undergoing above knee amputation | RCT | Pre-operative | 9 time points | 6 mo |
| **Johannesson 2010** | Outcomes of a standardized surgical and rehabilitation program in transtibial amputation for peripheral vascular disease: a prospective cohort study. | Cohort | Pre-operative | 4 time points | 12 mo |
| **Wilson 2008** | A randomised double blind trial of the effect of pre-emptive epidural ketamine on persistent pain after lower limb amputation. | RCT | Pre-operative | 5 time points | 12 mo |
| **Borghi 2010** | The use of prolonged peripheral neural blockade after lower extremity amputation: the effect on symptoms associated with phantom limb syndrome. | CrossSec | Pre-operative | 10 time points | 12 mo |
| **Norvell 2011** | Defining successful mobility after lower extremity amputation for complications of peripheral vascular disease and diabetes. | Cohort | Pre-operative | 4 time points | 12 mo |
| **Shalaeva 2017** | Tenfold risk increase of major cardiovascular events after high limb amputation with non-compliance for secondary prevention measures. | NonRandExp | Pre-operative | 7 time points | 12 mo |
| **Buchheit 2019** | The Impact of Surgical Amputation and Valproic Acid on Pain and Functional Trajectory: Results from the Veterans Integrated Pain Evaluation Research (VIPER) Randomized, Double-Blinded Placebo-Controlled Trial. | RCT | Pre-operative | 4 time points | 6 mo |
| **Williams 2014** | Changes in cognitive function from presurgery to 4 months postsurgery in individuals undergoing dysvascular amputation. | Cohort | Pre-operative | 3 time points | 4 mo |
| **Hunt 2023** | Effect of a continuous perineural levobupivacaine infusion on pain after major lower limb amputation: A randomised double-blind placebo-controlled trial | RCT | Pre-operative | 9 time points | 6 mo |
| **Webster 2012** | Prosthetic fitting, use, and satisfaction following lower-limb amputation: a prospective study. | Cohort | Pre-operative | 4 time points | 12 mo |
| **Rosenberg 2013** | Body mass index patterns following dysvascular lower extremity amputation. | Cohort | Pre-operative | 4 time points | 12 mo |
| **Turner 2014** | Prevalence and 1-year course of alcohol misuse and smoking in persons with lower extremity amputation as a result of peripheral arterial disease. | Cohort | Pre-operative | 4 time points | 12 mo |
| **Washington 2016** | An exploratory phenomenological study exploring the experiences of people with systemic disease who have undergone lower limb amputation and its impact on their psychological well-being. | QualRes | Pre-operative | 2 time points | 4 mo |
| **Czerniecki 2012** | Mobility changes in individuals with dysvascular amputation from the presurgical period to 12 months postamputation. | Cohort | Pre-operative | 4 time points | 12 mo |
| **Kohler 2011** | Feasibility of using a checklist based on the International Classification of Functioning, Disability and Health as an outcome measure in individuals following lower limb amputation. | Cohort | Pre-operative | 4 time points | 3 mo |
| **Fortington 2013** | Change in health-related quality of life in the first 18 months after lower limb amputation: a prospective, longitudinal study. | Cohort | Pre-operative | 3 time points | 18 mo |
| **LambertAw 2001** | Randomized prospective study comparing preoperative epidural and intraoperative perineural analgesia for the prevention of postoperative stump and phantom limb pain following major amputation. | RCT | Pre-operative | 7 time points | 12 mo |
| **Coffey 2014** | A prospective study of the importance of life goal characteristics and goal adjustment capacities in longer term psychosocial adjustment to lower limb amputation. | Cohort | Pre-operative | 2 time points | 6 mo |
| **Czerniecki 2012** | The effect of rehabilitation in a comprehensive inpatient rehabilitation unit on mobility outcome after dysvascular lower extremity amputation. | Cohort | Pre-operative | 4 time points | 12 mo |
| **O'Banion 2022** | Lower extremity amputation protocol: a pilot enhanced recovery pathway for vascular amputees | NonRandExp | Pre-operative | 9 time points | 3 mo |
| **Karanikolas 2011** | Optimized perioperative analgesia reduces chronic phantom limb pain intensity, prevalence, and frequency: a prospective, randomized, clinical trial. | RCT | Pre-operative | 6 time points | 6 mo |
| **Albright-Trainer 2022** | Peripheral nerve stimulation for the management of acute and subacute post-amputation pain: A randomized, controlled feasibility trial | RCT | Pre-operative | 5 time points | 3 mo |
| **SilvaADM 2021** | Functional capacity of elderly with lower-limb amputation after prosthesis rehabilitation: a longitudinal study. | NonRandExp | Pre-operative | 4 time points | 4 mo |
| **Godlwana 2020** | The effect of a home exercise intervention on persons with lower limb amputations: a randomized controlled trial | RCT | Pre-operative | 3 time points | 6 mo |
| **Roepke 2019** | A Prospective Longitudinal Study of Trajectories of Depressive Symptoms After Dysvascular Amputation. | Cohort | Pre-operative | 4 time points | 12 mo |
| **Einfeldt 2022** | Knee disarticulation following oncologic total knee arthroplasty: A 5-year follow-up case report. | CaseRpt | Pre-operative | 3 time points | 60 mo |
| **Ahmad 2022** | Use of popliteal brachial pressure index as a predictor of wound healing after below-knee amputations with absent popliteal pulse | Cohort | Pre-operative | 3 time points | 3 mo |
| **Akhtar 2022** | Osseointegration Following Transfemoral Amputation After Infected Total Knee Replacement: A Case Series of 10 Patients With a Mean Follow-up of 5 Years | Cohort | Pre-operative | at least 2 time points | 24 mo |
| **Henderson 2016** | Sexual activity after dysvascular lower extremity amputation. | Cohort | Pre-operative | 4 time points | 12 mo |
| **Williams 2014** | The role of expectations in pain after dysvascular lower extremity amputation. | Cohort | Pre-operative | 3 time points | 12 mo |
| **Toursarkissian 2002** | Major lower-extremity amputation: Contemporary experience in a single Veterans Affairs institution | Cohort | Pre-operative | 3 time points | 11 mo |
| **Peters 2019** | Is amputation in the elderly patient with critical limb ischemia acceptable in the long term? | Cohort | Pre-operative | 4 time points | 24 mo |
| **Wukich 2017** | Improved Quality of Life after Transtibial Amputation in Patients with Diabetes-Related Foot Complications | Cohort | Pre-operative | 2 time points | 12 mo |
| **Akhtar 2021** | Transtibial osseointegration for patients with peripheral vascular disease a case series of 6 patients with minimum 3-year follow-up | CaseRpt | Pre-operative | 3 time points | Mean follow-up of 48 mo |
| **MacCallum 2019** | Ambulatory Status following Major Lower Extremity Amputation | Cohort | Pre-operative | 3 time points | 12 mo |
| **vanEijk 2012** | Predicting prosthetic use in elderly patients after major lower limb amputation. | Cohort | Pre-operative | 2 time points | 12 mo |
| **Kamrad 2020** | Swedish Amputation and Prosthetics Registry: 8-year data on 5762 patients with lower limb amputation show sex differences in amputation level and in patient-reported outcome | Cohort | Pre-operative | 3 time points | 24 mo |
| **Callaghan 2008** | Using the common-sense self-regulation model to determine psychological predictors of prosthetic use and activity limitations in lower limb amputees. | Cohort | Pre-operative | 3 time points | 9 mo |
| **TenDuis 2009** | Knee disarticulation: survival, wound healing and ambulation. A historic cohort study. | Cohort | Post-operative | Not specific time points | 118.8 mo |
| **Singh 2012** | Effect of fluid collections on long-term outcome after lower limb amputation. | Cohort | Post-operative | 2 times points | 24 mo |
| **Hunter 2019** | Cognition Predicts Mobility Change in Lower Extremity Amputees Between Discharge from Rehabilitation and 4-Month Follow-up: A Prospective Cohort Study. | Cohort | Post-operative | 3 time points | 4 mo |
| **Davie-Smith 2019** | The Influence of Socio-economic Deprivation on Mobility, Participation, and Quality of Life Following Major Lower Extremity Amputation in the West of Scotland. | Cohort | Post-operative | 2 time points | 6 mo |
| **Christiansen 2018** | Behavior-Change Intervention Targeting Physical Function, Walking, and Disability After Dysvascular Amputation: A Randomized Controlled Pilot Trial. | RCT | Post-operative | 3 time points | 6 mo |
| **Chang 2021** | Targeted Muscle Reinnervation Improves Pain and Ambulation Outcomes in Highly Comorbid Amputees. | Cohort | Post-operative | 3 time points | 12 mo |
| **Couture 2011** | Cognitive appraisal and perceived benefits of dysvascular lower limb amputation: a longitudinal study. | Cohort | Post-operative | 3 time points | 3.5 mo |
| **Barnett 2013** | Postural responses during volitional and perturbed dynamic balance tasks in new lower limb amputees: a longitudinal study. | Cohort | Post-operative | 3 time points | 6 mo |
| **Prvu-Bettger 2009** | Short- and long-term prognosis among veterans with neurological disorders and subsequent lower-extremity amputation. | Cohort | Post-operative | 2 time points | 12 mo |
| **Madsen 2019** | Age and health-related quality of life, general self-efficacy, and functional level 12¬†months following dysvascular major lower limb amputation: a prospective longitudinal study. | Cohort | Post-operative | 4 times points | 12 mo |
| **Zidarov 2009** | Quality of life of persons with lower-limb amputation during rehabilitation and at 3-month follow-up. | Cohort | Post-operative | 3 time points | 3 mo |
| **Franchignoni 2003** | Is the Rivermead Mobility Index a suitable outcome measure in lower limb amputees? A psychometric validation study. | Cohort | Post-operative | 2 time points | not specified |
| **Wezenberg 2019** | Cardiorespiratory fitness and physical strain during prosthetic rehabilitation after lower limb amputation. | Cohort | Post-operative | at the start (T0) and end (T1) of the regular rehabilitation program | not specified |
| **Williams 2015** | Relationship between cognition and functional outcomes after dysvascular lower extremity amputation: a prospective study. | Cohort | Post-operative | 3 time points | 12 mo |
| **Schoppen 2003** | Physical, mental, and social predictors of functional outcome in unilateral lower-limb amputees. | Cohort | Post-operative | 4 time points | 12 mo |
| **Milosevic 2021** | Exploring patients’ experiences of analgesia after major lower limb amputation: a qualitative study | QualRes | Post-operative | 2 time points | 6 mo |
| **Mishra 2007** | Incidence and management of phantom limb pain according to World Health Organization analgesic ladder in amputees of malignant origin. | Cohort | Post-operative | 12 time points | 24 mo |
| **Roffman 2014** | Predictors of non-use of prostheses by people with lower limb amputation after discharge from rehabilitation: development and validation of clinical prediction rules | Cohort | Post-operative | 4 time points | 8 mo |
| **O‚ÄôBrien 2022** | Longitudinal durability of patient-reported pain outcomes after targeted muscle reinnervation at the time of major limb amputation | CaseRpt | Post-operative | 4 time points | 15 mo |
| **Norvell 2016** | The development and validation of a novel outcome measure to quantify mobility in the dysvascular lower extremity amputee: the amputee single item mobility measure | Cohort | Post-operative | 4 time points | 12 mo |
| **Livani 2011** | Sensate composite calcaneal flap in leg amputation: a full terminal weight-bearing surface-experience in eight adult patients | CaseRpt | Post-operative | 3 time points | 28.3 mo |
| **Brooks 2001** | The 2-minute walk test as a measure of functional improvement in persons with lower limb amputation | Cohort | Post-operative | 3 time points | 3 mo |
| **Vrieling 2009** | Gait adjustments in obstacle crossing, gait initiation and gait termination after a recent lower limb amputation | Cohort | Post-operative | 4 time points | 6 mo |
| **Nehler 2003** | Functional outcome in a contemporary series of major lower extremity amputations | Cohort | Post-operative | 4 time points | 17 mo |
| **Sauter 2012** | Effect of post-acute rehabilitation setting on functional outcomes of persons undergoing dysvascular lower extremity amputations | Cohort | Post-operative | 2 time points | 6 mo |
| **Chopra 2018** | Ambulation and functional outcome after major lower extremity amputation | Cohort | Post-operative | Exact time points are not specified. | Mean follow-up of 178.7 mo |
| **Guo 2021** | Contralateral Acupuncture for the Treatment of Phantom Limb Pain and Phantom Limb Sensation in Oncologic Lower Limb Amputee: A Case Report | CaseRpt | Post-operative | 2 time points | 9 mo |
| **Wong 2023** | Beyond exercise. Can application of manual therapy before exercise benefit a low functioning person with limb loss? A case study. | CaseRpt | Post-operative | 3 time points | 3 mo |
| **Dite 2007** | Clinical identification of multiple fall risk early after unilateral transtibial amputation. | Cohort | Post-operative | 2 time points | 6 mo |
| **Purushothaman 2023** | Assessment of efficiency of mirror therapy in preventing phantom limb pain in patients undergoing below-knee amputation surgery-a randomized clinical trial. | RCT | Post-operative | 4 time points | 6 mo |
| **Coffey 2014** | Goal pursuit and goal adjustment as predictors of disability and quality of life among individuals with a lower limb amputation: a prospective study. | Cohort | Post-operative | 3 time points | 6 mo |
| **Anderson 2017** | Perceived social support moderates the relationship between activities of daily living and depression after lower limb loss.C44 | Cohort | Post-operative | 2 time points | 12 mo |
| **Roepke 2017** | A Longitudinal Study of Social Participation After Dysvascular Lower Extremity Amputation. | Cohort | Post-operative | 4 times points | 12 mo |
| **Giuffrida 2010** | Contralateral stimulation, using TENS, of phantom limb pain: two confirmatory cases. | CaseRpt | Post-operative | 7 time points | 12 mo |
| **Mitton 2017** | Fluctuating residual limb volume accommodated with an adjustable, modular socket design: A novel case report. | CaseRpt | Post-operative | 5 time points | 9.5 mo |
| **Pezzin 2013** | Effect of postacute rehabilitation setting on mental and emotional health among persons with dysvascular amputations. | Cohort | Post-operative | 2 time points | 6 mo |
| **Coffey 2014** | Goal management tendencies predict trajectories of adjustment to lower limb amputation up to 15 months post rehabilitation discharge. | Cohort | Post-operative | 4 time points | 15 mo |
| **Remes 2009** | Predictors for institutionalization and prosthetic ambulation after major lower extremity amputation during an eight-year follow-up. | Cohort | Post-operative | 3 time points | 24 mo |
| **Rostaminejad 2017** | Efficacy of eye movement desensitization and reprocessing on the phantom limb pain of patients with amputations within a 24-month follow-up. | RCT | Post-operative | 3 times points | 24 mo |
| **Remes 2010** | Quality of life three years after major lower extremity amputation due to peripheral arterial disease. | CaseCtrl | Prosthetic | 2 time points | 32.4 mo |
| **Franchignoni 2020** | The minimal clinically important difference of the Prosthesis Evaluation Questionnaire - Mobility Scale in subjects undergoing lower limb prosthetic rehabilitation training. | Cohort | Prosthetic | 2 time points | about 3 to 6 mo |
| **Rommers 2000** | A study of technical changes to lower limb prostheses after initial fitting. | Cohort | Prosthetic | 3 time points | 12 mo |
| **Desrochers 2019** | Relationship between body image and physical functioning following rehabilitation for lower-limb amputation. | Cohort | Prosthetic | 2 time points | 4 mo |
| **Lass 2013** | The effect of a flexible pylon system on functional mobility of transtibial amputees. A prospective randomized study. | RCT | Prosthetic | 3 time points | at least 6 mo |
| **Franchignoni 2019** | Sensitivity to change and minimal clinically important difference of the Locomotor Capabilities Index-5 in people with lower limb amputation undergoing prosthetic training. | Cohort | Prosthetic | 2 time points | not specified |
| **Hoffstad 2015** | Diabetes, Lower-Extremity Amputation, and Death | Cohort | Life-long care/maintenance | 4 time points | 107 mo |

- **RCT** – Randomized controlled trial; experimental design with random assignment.
- **CaseRpt** – Case report/series; descriptive report(s) of individual or small groups of patients.
- **NonRandExp** – Non-randomized experimental study; experimental design without randomization.
- **QualRes** – Qualitative research (interview study); interview-based or narrative qualitative design.
- **CrossSec** – Cross-sectional study (observational); observational data collected at a single time point.
- **CaseCtrl** – Case control study (comparing patients to controls, usually no intervention); observational design comparing groups based on outcome status.
- **Mo** – Month

Appendix IV: Complete outcome measure table

*Note: most of the outcome measures has been used in several studies (as the count number indicates), we only mentioned one of the corresponding references.*

| **Type** | **Outcome measure** | **Count** | **Corresponding Reference** |
| --- | --- | --- | --- |
| **Cognitive** | Validity of Cognition (VoC) | 1 | Rostaminejad 2017 |
|  | Trail Making Test (TMT) | 1 | Hunter 2019 |
|  | Single Task Cognitive Assessment (serial 3s) | 1 | Hunter 2019 |
|  | Short Portable Mental Status Questionnaire (SPMSQ) | 3 | Williams 2014 |
|  | Neuropsychological Status | 1 | Williams 2015 |
|  | Montreal Cognitive Assessment (MoCA) | 1 | Hunter 2019 |
|  | Mini Mental State Examination (MMSE) | 3 | Remes 2009 |
|  | Clifton Assessment Procedures for the Elderly (CAPE) | 1 | Callaghan 2008 |
| **Demographic** | Type of Residence Before Amputation | 1 | Johannesson 2010 |
|  | Office of Population Censuses and Surveys Scale (OPCS) | 1 | Panesar 2001 |
| **Financial** | Average Costs of Prosthetics | 1 | Rommers 2000 |
| **Medical** | Veterans Administration Large Health Survey | 2 | Czerniecki 2012 |
|  | Time of First Dose of Analgesia After Surgery | 1 | Purushothaman 2023 |
|  | The Barthel Index of Activities of Daily Living | 5 | Rommers 2000 |
|  | Rehabilitation Environment Assessment | 1 | Czerniecki 2012 |
|  | Re-amputation or Surgery | 1 | Johannesson 2010 |
|  | Presence of Other Diseases, Comorbidities ICD10 | 8 | Ahmad 2022 |
|  | Postoperative Length of Stay (LOS) | 4 | O'Banion 2022 |
|  | Popliteal Pressure | 1 | Ahmad 2022 |
|  | Popliteal Brachial Index | 1 | Ahmad 2022 |
|  | Opioid Consumption | 1 | Albright-Trainer 2022 |
|  | Number of Rehabilitation Therapy Visits by Environment of Care | 1 | Czerniecki 2012 |
|  | Number of Analgesic /Drugs Prescribed | 6 | Karanikolas 2011 |
|  | Major Adverse Cardiovascular Events (MACE) | 1 | Karanikolas 2011 |
|  | Hypertension | 1 | Ahmad 2022 |
|  | Healing and Wound Healing Time /Rate and Wound Management | 9 | Ahmad 2022 |
|  | Fluid Collection | 1 | Singh 2012 |
|  | Elixhauser Comorbidity Measure | 1 | Prvu-Bettger 2009 |
|  | Duration of Surgery | 1 | Prvu-Bettger 2009 |
|  | Different Blood Cell Counts | 5 | Chopra 2018 |
|  | Diabetes Miletus | 1 | Ahmad 2022 |
|  | Coronary Computed Tomographic Angiography (CCTA) | 1 | Shalaeva 2017 |
|  | Coronary Artery Calcium Score (CACS) | 1 | Shalaeva 2017 |
|  | Complications | 5 | Toursarkissian 2002 |
|  | Charlson Comorbidity Index | 6 | Hoffstad 2015 |
|  | Brachial Pressure | 1 | Ahmad 2022 |
|  | Blood Loss | 1 | Purushothaman 2023 |
|  | Ankle Brachial Index | 1 | Chopra 2018 |
|  | Smoking status | 1 | Webster 2012 |
|  | Alcohol Use Disorders Identification Test-Consumption (AUDIT-C) | 4 | Webster 2012 |
|  | Discharge Disposition | 2 | O'Banion 2022 |
| **Pain** | Visual Analog Scale (VAS) | 10 | Bakeer 2023 |
|  | Self-Reported Leeds Assessment of Neuropathic Symptoms and Signs Tool (S-LANSS) | 1 | Buchheit 2019 |
|  | Satisfaction with pain | 1 | Williams 2014 |
|  | Response to Pain Relief | 1 | Rostaminejad 2017 |
|  | Question on Amputation Related/Phantom Limb Pain and Frequency of Pain | 10 | LambertAw 2001 |
|  | Patient-Reported Outcomes Measurement Information System (PROMIS) Pain Interference Questionnaire Short-Form 8a | 1 | O‚ÄôBrien 2022 |
|  | Patient-Reported Outcomes Measurement Information System (PROMIS) Pain Behavior Questionnaire Short-Form 7a | 1 | O‚ÄôBrien 2022 |
|  | Patient Global Impression of Change | 3 | Albright-Trainer 2022 |
|  | Pain Interference (PI) Scores | 1 | Albright-Trainer 2022 |
|  | Pain Intensity | 1 | Karanikolas 2011 |
|  | Pain Expectation | 1 | Williams 2014 |
|  | Pain Disability Index | 1 | Albright-Trainer 2022 |
|  | Pain Catastrophizing Scale | 1 | Albright-Trainer 2022 |
|  | Number of Pain Location | 1 | Williams 2014 |
|  | NRS | 8 | O‚ÄôBrien 2022 |
|  | Neuropathic Pain Symptom Inventory (NPSI) | 1 | Guo 2021 |
|  | Neuropathic Pain Scale | 1 | Wilson 2008 |
|  | McGill Pain Questionnaire | 6 | Hunt 2023 |
|  | Effectiveness of Post-op Analgesia and Changes in Sensory Processing (QST) | 1 | Wilson 2008 |
|  | Defense and Veterans Pain Rating Scale (DVPRS) | 1 | Buchheit 2019 |
|  | Chronic Pain Grade | 1 | Henderson 2016 |
|  | Cambridge Phantom Limb Profile | 1 | Henderson 2016 |
|  | Budapest Clinical Criteria | 1 | Buchheit 2019 |
|  | Brief Pain Inventory Short Form and Original | 5 | Albright-Trainer 2022 |
|  | 5-Point Verbal Stump Pain Rating Scale | 2 | Borghi 2010 |
| **PB-Physical** | Timed Up and Go (TUG) | 8 | Godlwana 2020 |
|  | Walking Speed | 2 | Lass 2013 |
|  | VO peak | 1 | Wezenberg 2019 |
|  | Time to Receipt of Prosthesis | 1 | Johannesson 2010 |
|  | Time to Ambulation with Prosthesis | 1 | Johannesson 2010 |
|  | Limits of Stability Test | 1 | Barnett 2013 |
|  | 10-Minute Walk Test (TWT) | 1 | Franchignoni 2003 |
|  | Symmetry Index | 1 | Einfeldt 2022 |
|  | Success Rate in Obstacle Crossing | 1 | Vrieling 2009 |
|  | Step Count, Step Length | 3 | Wong 2023 |
|  | Sit-to-Stand Test | 1 | Wong 2023 |
|  | Sensory Organization Test | 1 | Barnett 2013 |
|  | Respiratory Exchange Ratio (RERpeak) | 1 | Wezenberg 2019 |
|  | Peak Power Output | 1 | Wezenberg 2019 |
|  | Peak Heart Rate | 1 | Wezenberg 2019 |
|  | Mortality/Survival | 11 | Hoffstad 2015 |
|  | Lower Limb Joint Angles | 1 | Vrieling 2009 |
|  | L Test of Functional Mobility | 2 | Desrochers 2019 |
|  | K-Levels | 2 | Akhtar 2022 |
|  | ICF Qualifier Scores | 1 | Kohler 2011 |
|  | Ground Reaction Force (and all the properties) | 7 | Vrieling 2009 |
|  | Generic Scale of Qualifiers | 1 | Kohler 2011 |
|  | Gait Velocity | 3 | Desrochers 2019 |
|  | Gait disorder | 1 | Lass 2013 |
|  | Functional Autonomy Measurement System (SMAF) | 1 | Couture 2011 |
|  | Functional Ambulation Categories (FAC) | 2 | VanEijk 2012 |
|  | Four Square Step Test (FSST) | 1 | Dite 2007 |
|  | Center of Pressure Shift | 1 | Vrieling 2009 |
|  | Borg Rating of Perceived Exertion | 1 | Wezenberg 2019 |
|  | BMI | 2 | Chopra 2018 |
|  | Berg Balance Scale (BBS) | 1 | Wong 2023 |
|  | 6 -Minutes-Walk Test | 1 | Akhtar 2021 |
|  | 2-Minute Walk Test | 3 | Brooks 2001 |
|  | 180 Degrees Turn Test | 1 | Dite 2007 |
| **PR-Physical** | World Health Organization Disability Assessment Schedule 2.0 (WHODAS 2.0) (also in PAR category) | 2 | Coffey 2014 |
|  | Weight-Bearing Ability | 1 | Livani 2011 |
|  | Walking Ability Index (WAI) | 1 | Rommers 2000 |
|  | Use of Prosthesis Time and Score | 7 | Nehler 2003 |
|  | Type of Prosthetic and Prosthetic Component / Level of Amputation | 2 | Rommers 2000 |
|  | Socket Comfort Score | 1 | Mitton 2017 |
|  | Sickness Impact Profile (SIP-68) | 1 | Schoppen 2003 |
|  | Rivermead Mobility Index (RMI) | 1 | Franchignoni 2003 |
|  | Question on Prosthetic Fitting | 3 | Norvell 2016 |
|  | Question on Mobility and Ambulatory Status and Daily Activity | 11 | Norvell 2016 |
|  | Question on Mobility Aid | 3 | Roffman 2014 |
|  | Question on Independence Donning/Doffing | 1 | Nehler 2003 |
|  | Question on Functional Status | 1 | Sauter 2012 |
|  | QTFA (also in PAR category) | 1 | Akhtar 2021 |
|  | Prosthetic Limb User Survey of Mobility (PLUS-M) | 1 | Davie-Smith 2019 |
|  | Pfeffer Questionnaire | 1 | SilvaADM 2021 |
|  | Number of Visits to Prosthetic Clinics | 1 | Rommers 2000 |
|  | Locomotor Capabilities Index (Modified and Original) | 16 | Norvell 2016 |
|  | Intensity Time in Activity | 1 | Christiansen 2018 |
|  | Houghton Scale | 1 | Brooks 2001 |
|  | Groningen Activity Restriction Scale (GARS) | 3 | Anderson 2017 |
|  | Fuster-BEWAT | 1 | Shalaeva 2017 |
|  | Functional Measure for Amputees (FMA) | 1 | Callaghan 2008 |
|  | Frenchay Activities Index (FAI) | 1 | vanEijk 2012 |
|  | FIM | 2 | Albright-Trainer 2022 |
|  | Fall events | 1 | Chopra 2018 |
|  | FAAM | 1 | Wukich 2017 |
|  | Amputee Activity Score (AAS) | 1 | Panesar 200 |
|  | ABC (Activities-Specific Balance Confidence) | 2 | Wong 2023 |
| **Psychological** | Trinity Amputation and Prosthesis Experience Scales TAPES (complete or subscale) (also in PAR category) | 4 | Norvell 2016 |
|  | The Groningen Questionnaire: Problems After Arm Amputation (GQPAA) | 1 | Buchheit 2019 |
|  | Tenacious Goal Pursuit Scale (TGP) | 2 | Coffey 2014 |
|  | Subjective Units of Distress Scale (SUDS) | 2 | Rostaminejad 2017 |
|  | Special Interest Group in Amputee Medicine (SIGAM) | 2 | Mitton 2017 |
|  | Social Support Survey (MSSS) Modified and Original | 6 | Remes 2009 |
|  | Six-Item Brief Social Support Questionnaire (SSQ6) | 1 | Remes 2010 |
|  | Self-reported Life Satisfaction Score (LS) | 1 | Remes 2010 |
|  | Satisfaction with Mobility | 1 | Norvell 2016 |
|  | Satisfaction with Life Scale (SWLS) | 1 | Norvell 2011 |
|  | Reintegration to Normal Living Index (RNLI) (also in PAR category) | 1 | Davie-Smith 2019 |
|  | Question on Cognitive Appraisal of the Amputation | 1 | Couture 2011 |
|  | Prosthetic Adaptation | 1 | Livani 2011 |
|  | Positive and Negative Affect Schedule | 2 | Coffey 2014 |
|  | Patient Health Questionnaire (PHQ-9) | 7 | Webster 2012 |
|  | Participation Scale | 1 | Williams 2015 |
|  | Multidimensional Scale of Perceived Social Support | 1 | Webster 2012 |
|  | Major Depressive Episode | 1 | Williams 2014 |
|  | Late Life Disability Index (also in PAR category) | 1 | Hunt 2023 |
|  | Illness Perception Questionnaire - Revised (IPQ-R) and original | 1 | Callaghan 2008 |
|  | HADS (Hospital Anxiety and Depression Scale) | 2 | Wilson 2008 |
|  | Groningen Questionnaire Problems Leg Amputation (GQPLA) | 1 | Buchheit 2019 |
|  | Goal Facilitation Inventory | 1 | Coffey 2014 |
|  | Goal Adjustment Scale | 1 | Coffey 2014 |
|  | Geriatric Depression Scale Short Form 15 (GDS) | 2 | Pezzin 2013 |
|  | General Self-Efficacy (GSE) | 1 | Madsen 2019 |
|  | Flexible Goal Adjustment Scale (FGA) | 2 | Coffey 2014 |
|  | Craig Handicap Assessment and Reporting Technique (also in PAR category) | 1 | Williams 2015 |
|  | Community Integration Questionnaire (also in PAR category) | 2 | Williams 2015 |
|  | Center of Epidemiological Studies Depression Scale (CES-D) | 1 | Peters 2019 |
|  | Beck Depression Inventory | 2 | Coffey 2014 |
|  | Amputee Body Image Scale (ABIS) | 2 | Desrochers 2019 |
| **Quality of life** | World Health Organization Quality of Life Scale Brief Version (WHO-QOL-BREF) (also in PAR category) | 2 | Coffey 2014 |
|  | The Euroqol-5D | 3 | Godlwana 2020 |
|  | SF-36 (also in PAR category) | 10 | Brooks 2001 |
|  | SF-12 (also in PAR category) | 1 | Peters 2019 |
|  | Semi-Structured Interviews | 5 | Washington 2016 |
|  | RAND-36 (also in PAR category) | 1 | Fortington 2013 |
|  | Question on the Importance of Sexual Activity | 1 | Henderson 2016 |
|  | Question on Sexual Activity | 1 | Henderson 2016 |
|  | Question on Quality of Life | 1 | Roepke 2019 |
|  | Question on Perceived General Health | 2 | Roepke 2019 |
|  | Question on Desire for Sexual Activity | 1 | Henderson 2016 |
|  | PEQ | 4 | Wong 2023 |
|  | 15D Health Related QoL scale (15D HRQoL) | 1 | Kamrad 2020 |
| **Grand Total** |  | **174** |  |

PB-PHY= Performance- based physical outcome measures

PR-PHY= Patient- reported physical outcome measures

PAR = Partially covers Participation (at least one question related to participation)
